# Exposome variations affect *Drosophila* bristle patterning via the regulation of proneural genes through distinct mechanisms

**DOI:** 10.1038/s41598-025-20122-6

**Published:** 2025-10-16

**Authors:** Valérie Ribeiro, Nicolas Doucet, Michel Gho, Agnès Audibert, Jean-Michel Gibert

**Affiliations:** 1 Development, Adaptation and Ageing, Dev2A, CNRS, Inserm, Sorbonne Université, 75005 Paris, France; 2 CNRS, Institut de Biologie Paris-Seine, IBPS, Inserm, Sorbonne Université, 75005 Paris, France

**Keywords:** Developmental biology, Genetics, Ecology, Environmental sciences

## Abstract

How developing organisms respond to a changing environment is a fundamental question. Pollutants and temperature are major environmental factors. Using the bristle patterning of *Drosophila* as a model system, we observed that cold temperature and methotrexate, a medical drug that contaminates wastewaters, increase dorsocentral (DC) bristle number*,* a trait normally robust. The patterning of bristles is well understood and involves the *achaete-scute (ac-sc)* proneural genes. Modular enhancers activate *ac-sc* expression in groups of cells, called proneural clusters, from which bristle precursors are selected by lateral inhibition, a process involving Notch signalling and *ac-sc* auto-activation. In addition, *ac-sc* basal expression is controlled by a cocktail of repressive factors. We observed that the deletion of the DC enhancer prevents the induction of ectopic DC bristles by methotrexate but does not stop low temperature to induce DC bristles. Indeed, we show that methotrexate has a strong synergy with mutants of factors that regulate the DC enhancer and extends the zone of activity of this enhancer. In contrast, temperature interacts with repressors of *ac-sc* basal expression. Thus, methotrexate and temperature both affect DC bristle patterning but by distinct mechanisms, methotrexate on the DC enhancer and cold independently of this enhancer.

**Supplementary Information:**

The online version contains supplementary material available at 10.1038/s41598-025-20122-6.

## Introduction

Organism development produces a nearly constant phenotype despite genetic variations, environmental fluctuations and the stochasticity of molecular and cellular processes. This property of development is commonly referred to as robustness^1,2^. In an era of anthropogenic changes such as pollution or global warming, it is important to analyse how the exposome, which corresponds to the totality of environmental factors to which an organism is expose during its lifetime^3^, challenges developmental robustness.

Robustness must also be flexible enough to allow phenotypes to evolve. The challenge of developmental robustness by environmental change can facilitate evolution. Indeed, environmental change can induce phenotypic variation on which selection can act. Thanks to the selection of particular genetic variants, the phenotype initially observed only under certain environmental conditions becomes constitutive and independent of the environment. This process was discovered by Waddington using experimental populations of *Drosophila* and was named genetic assimilation^4–6^. Some empirical studies show that this is not limited to laboratory experiments but that such modes of evolution, called Plasticity-first-evolution or Flexible-stem-hypothesis, also occur in the wild^7–13^. Thus, the environment can influence evolution not only as a selective agent but also, in some cases, as an initiator of phenotypic variation. It is therefore essential to analyse how developmental systems respond to environmental changes.

In adult drosophilids, large mechanosensory bristles (macrochaetes) occupy stereotyped positions and exhibit species-specific robust patterns used in systematics (chaetotaxy)^14^. In the model species *Drosophila melanogaster*, two pairs of macrochaetes, the anterior and posterior dorsocentral bristles (DC bristles), are located on the mesonotum, the dorsal sclerite of the second thoracic segment. This pattern of two pairs of DC bristles is primitive and widely observed in drosophilids^14^. It is also observed in the oldest known drosophilid species, *Electrophortica succini*, from the Eocene Baltic amber^15^, 34–48 million years old^16^. However, this pattern has been modified in a few drosophilid lineages. For example, the anterior DC bristles have been lost in the lineages of *Pseudostegana, Drosophila (Dichaetophora) abberans, Mycodrosophila dimidiata* and *Baeodrosophila pubescens*^14^. By contrast, in a few other lineages, the number of DC bristles is increased and additional bristles are located more anteriorly on the mesonotum. This is a feature of isolated species or a shared derived character (synapomorphy) of a particular group of species. For example, the *Drosophila polychaeta* species group is characterised by three pairs of DC bristles^17^ and the *Drosophila quadrisetata* group by four pairs of DC bristles^18^. In the *Drosophila quadrilineata* species group, the number of pairs of DC bristles varies from two to four^19,20^. The increase in DC bristle number in the *Drosophila quadrilineata* lineage has been shown to involve changes in an enhancer of the *achaete-scute* proneural genes^20^, but the early steps in this morphological evolution and the initial role of the environment are not known. Certain environmental variations may have facilitated the evolution of the DC bristle pattern by revealing cryptic genetic variation^21^. For example, it has been shown that low temperature increases the number of ectopic DC bristles in lines of *Drosophila melanogaster* artificially selected for high numbers of these bristles^22^. In any case, the robustness of DC bristle development has been broken in several drosophilid lineages allowing the exploration of a diversified morphospace. This robust but evolvable pattern of two pairs of DC bristle represents an interesting model to analyse how the exposome affects development, especially since the mechanisms involved in the patterning of these bristles are well understood^23^.

The proneural genes *achaete (ac)* and *scute (sc)* play an essential role in the establishment of the thoracic bristle pattern. They are expressed in the presumptive notum in the wing imaginal discs of third instar larvae in groups of cells called proneural clusters from which bristle precursors are selected by lateral inhibition, a process involving the Notch signalling pathway and the auto-activation of *ac-sc*^24,25^. Lateral inhibition progressively restricts *ac-sc* expression to bristle precursors. The modular enhancers that activate *ac-sc* expression in the wing imaginal disc proneural clusters are scattered over the 100 kb of the *ac-sc* complex^25^. The enhancer that activates *ac-sc* expression in the DC proneural cluster, the DC enhancer, is located around 4 kb upstream of *ac* and is directly activated by the GATA transcription factor Pannier (Pnr)^26^. *pnr* is expressed in a dorsal longitudinal band and *u-shaped (ush)* encoding a Friend of GATA factor is expressed within the *pnr* domain. Ush and Pnr form a heterodimer that represses the DC enhancer^26,27^. This restricts the activity of the DC enhancer along the dorsolateral axis to the region where *pnr* is expressed but not *ush*. In addition, Chip links Pnr bound to the DC enhancer to Ac-Sc/Daughterless heterodimers bound to *ac-sc* promoters^28^. This enhances *ac-sc* expression in the DC proneural cluster. The demarcation of DC enhancer activity along the antero-posterior axis is less well understood but involves the morphogen Dpp which signals from the posterior region of the presumptive notum^29^. In addition to the transcriptional activation of *ac-sc* in proneural clusters by specific enhancers, there is also a basal expression of *ac-sc* that is controlled by a cocktail of repressors including Hairy (Hry), Extra macrochaetae (Emc), Stripe (Sr) and Poils au dos (Pad)^30,31^. Some of them such as Hry are direct repressors of *ac-sc* transcription^32^, whereas others such as Emc affect *ac-sc* post-transcriptionally and control *ac-sc* basal level by interfering with *ac-sc* auto-activation^33^. In mutants for these repressors, an increase in *ac-sc* expression leads to the formation of ectopic bristle precursors outside the regions of activity of proneural cluster enhancers^30,31^. Thus, independent mechanisms are required in parallel to produce the wild-type bristle pattern. It follows that DC bristle number can be increased by enhancing *ac-sc* expression in several ways: first, by increasing the activity of the DC enhancer; second, by increasing the basal expression of *ac-sc;* third, by reducing the level of lateral inhibition.

Here we observe that two environmental factors, low developmental temperature and the drug methotrexate increase the number of DC bristles in a wild-type stock. Temperature, a critical abiotic factor, has a major impact on living organisms, especially ectotherms, and shapes the geographical distribution of many species^34^. Methotrexate is a folic acid analogue used in high doses to treat various cancers and in low doses to treat certain autoimmune diseases (psoriasis, Crohn’s disease, rheumatoid arthritis)^35^. The high doses at which methotrexate is used raise environmental concerns because it was shown to contaminate hospital staff and to be present in the influent and effluent of sewage treatment plants at concentrations with biological impact^36–40^. We use the genetic tools available in *D. melanogaster* to dissect the mechanisms by which these factors affect DC bristle development and show that methotrexate induces the formation of ectopic DC bristles via an enlargement of the zone of activity of the DC enhancer, whereas low temperature does so independently of this enhancer by interacting with repressors of *ac-sc* basal level.

## Results

### Low temperature and methotrexate induce ectopic DC bristles

**Fig. 1:**
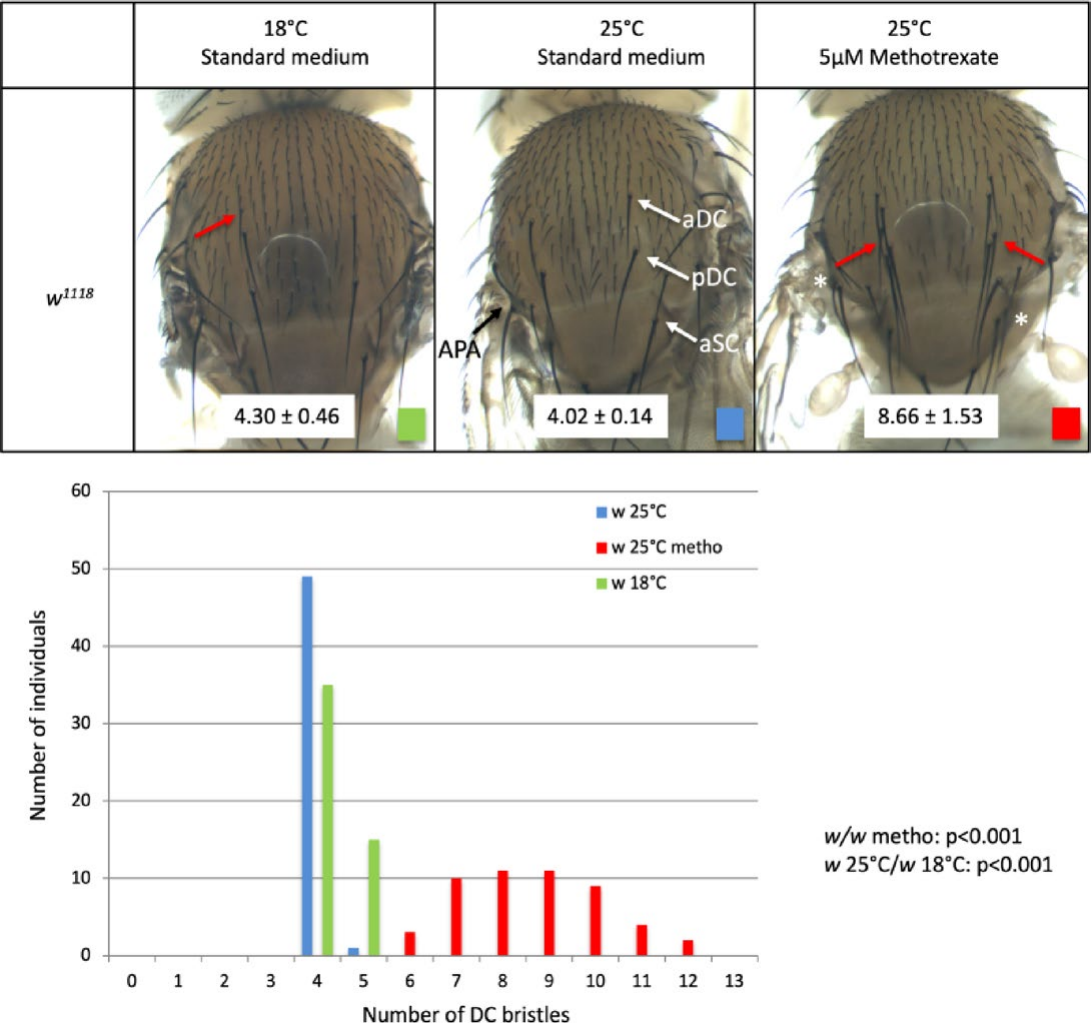
Low temperature and methotrexate induce ectopic DC bristles. Upper part: DC bristle phenotypes of a wild type line (*w*^*1118*^) grown on standard medium at 25 °C (middle), on standard medium at 18 °C (left) or on 5 µM methotrexate at 25 °C (right). In the middle panel, anterior and posterior DC bristles are indicated by white arrows (aDC and pDC respectively). The anterior scutellar (aSC) and anterior post-alar (APA) bristles are indicated by white and black arrows respectively. In the left panel, an ectopic DC bristle induced at 18 °C is indicated by a red arrow. In the right panel, many ectopic DC bristles are observed on 5 µM methotrexate (red arrows) and left APA and right aSC are duplicated in this individual (white asterisks). Mean DC bristle number and standard deviations are indicated on a white background on the pictures (N = 50 for each condition). Lower part: graphical representation of the data (number of individuals per number of DC bristles for each condition).

We observed that low temperature (18 °C instead of 25 °C) and the addition of methotrexate to the larval diet at 5 µM increased DC bristle number (Fig. 1). Flies developed at 25 °C on standard medium carry 4.02 ± 0.14 DC bristles (blue, N = 50), whereas flies developed at 18 °C on standard medium carry 4.3 ± 0.46 DC bristles (green, Wilcoxon rank sum test *p* < 0.001; N = 50) and flies developed at 25 °C on 5 µM methotrexate carry 8.66 ± 1.53 DC bristles (red, Wilcoxon rank sum test *p* < 0.001; N = 50). Low temperature induces the formation of ectopic bristles located anteriorly to the anterior DC bristles in most cases (13/15). Methotrexate leads to the formation of ectopic bristles around both anterior and posterior DC bristle normal positions. We noticed that methotrexate induces also the formation of ectopic bristles located in other regions of the thorax. Indeed, duplicated anterior scutellar bristles and anterior postalar bristles are occasionally observed for example (Fig. 1, asterisks). However, due to their stronger response and the knowledge on their development, we centred our analysis on DC bristles. This increase in mean DC bristle number by low temperature and methotrexate is also associated with a significant increase in its variance. Indeed, DC bristle number variance in standard condition (V = 0.02) is significantly different from the variances of this trait at 18 °C (V = 0.214, Levene test *p* < 0.0001) or on 5 µM methotrexate (V = 2.351, Levene test *p* < 0.0001). Thus, low temperature and methotrexate affect DC bristle development and its robustness.

### Deletion of the DC enhancer prevents the effect of methotrexate on DC bristle development but does not stop low temperature to induce ectopic DC bristles

**Fig. 2:**
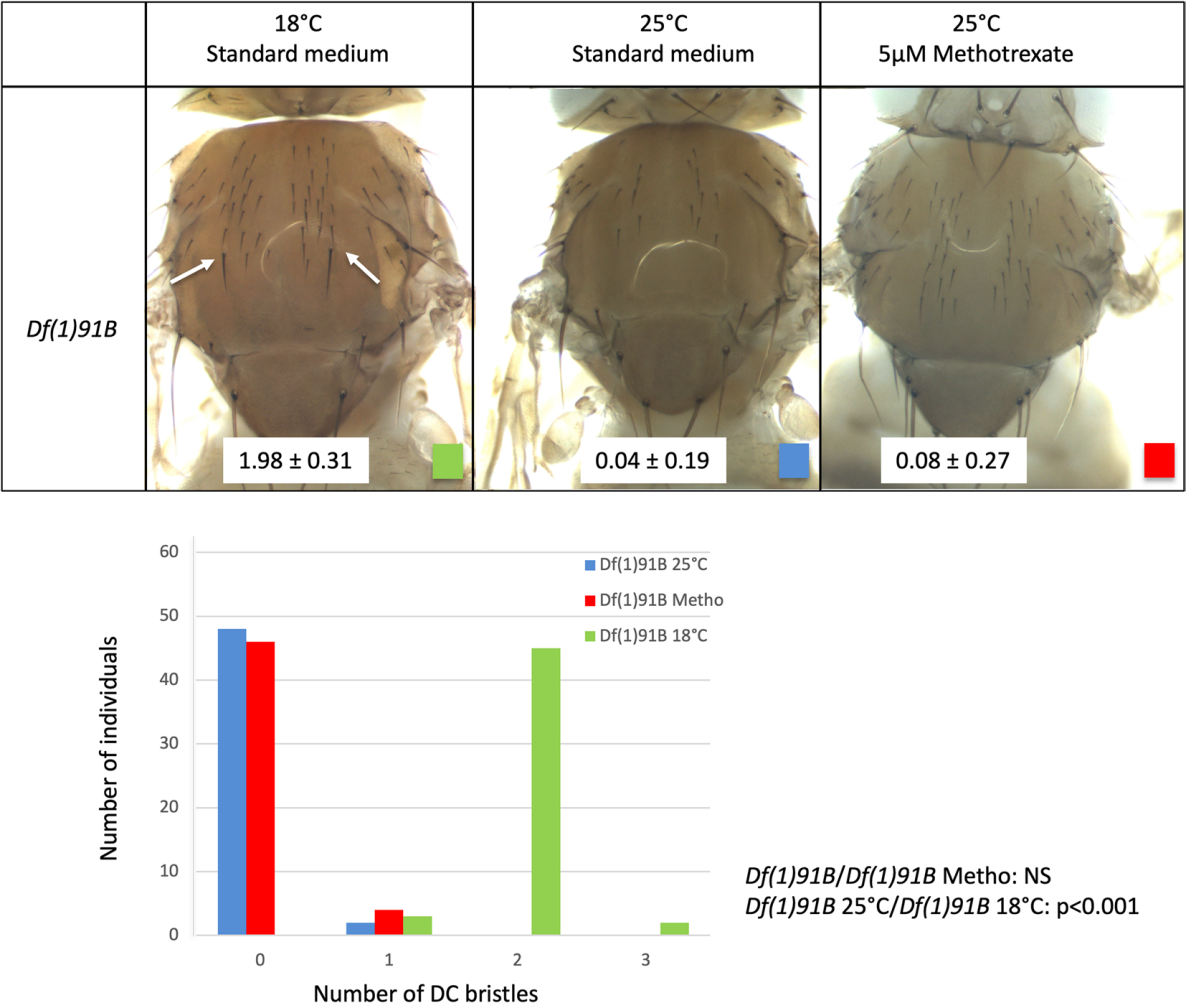
The DC enhancer is required for the effect of methotrexate but not that of cold. Upper part: DC bristle phenotypes of the line *Df(1)91B* carrying a deletion of *ac-sc* DC enhancer grown in the same environmental condition as Fig. 1. Almost no DC bristles are formed on standard conditions or on 5 µM methotrexate at 25 °C. By contrast, 2 DC bristles develop in most individuals at 18 °C on standard medium (white arrows). Mean DC bristle number and standard deviations are indicated on a white background on the pictures (N = 50 for each condition). Lower part: graphical representation of the data (number of individuals per number of DC bristles for each condition).

The consequence of low developmental temperature and methotrexate on DC bristle development prompted us to test whether *ac-sc* DC enhancer was required for these effects. To do so, we used the deficiency *Df(1)91B* which deletes this enhancer^30^ and abolish *sc* expression in the DC proneural cluster^20^ (Fig. 2). In this mutant background, at 25 °C, DC bristles were absent except for a minority of flies carrying a single remaining bristle in the region of the anterior DC bristles (blue, 0.04 ± 0.20 DC bristles, N = 50). We found that when *Df(1)91B* flies are grown on 5 µM methotrexate at 25 °C, the number of DC bristles was not significantly modified (red, 0.08 ± 0.27 DC bristles, N = 50, Wilcoxon rank sum test *p* = 0.407). By contrast, when *Df(1)91B* flies were grown on standard medium at 18 °C, the number of DC bristles significantly increased: the majority of individuals had two bristles in the region carrying anterior DC bristles in wild-type flies (green, white arrows, 1.98 ± 0.32 DC bristles, N = 50, Wilcoxon rank sum test *p* < 0.001). Interestingly, it is in this area that most ectopic DC bristles form in *w*^*1118*^ flies grown at 18 °C.

Thus, the DC enhancer is required for the effect of methotrexate on DC bristle development, whereas low temperature seems to affect DC bristle patterning independently of this enhancer.

### Factors regulating the DC enhancer and methotrexate show strong genotype by environment interactions

The requirement of the DC enhancer for the effect of methotrexate on DC bristle development led us to test whether sensitizing the genetic background with mutations for DC enhancer regulators would affect the effect of methotrexate. To do so, we analysed the interactions (non-additive effects) between this drug and genes encoding regulators of the DC enhancer.

**Fig. 3:**
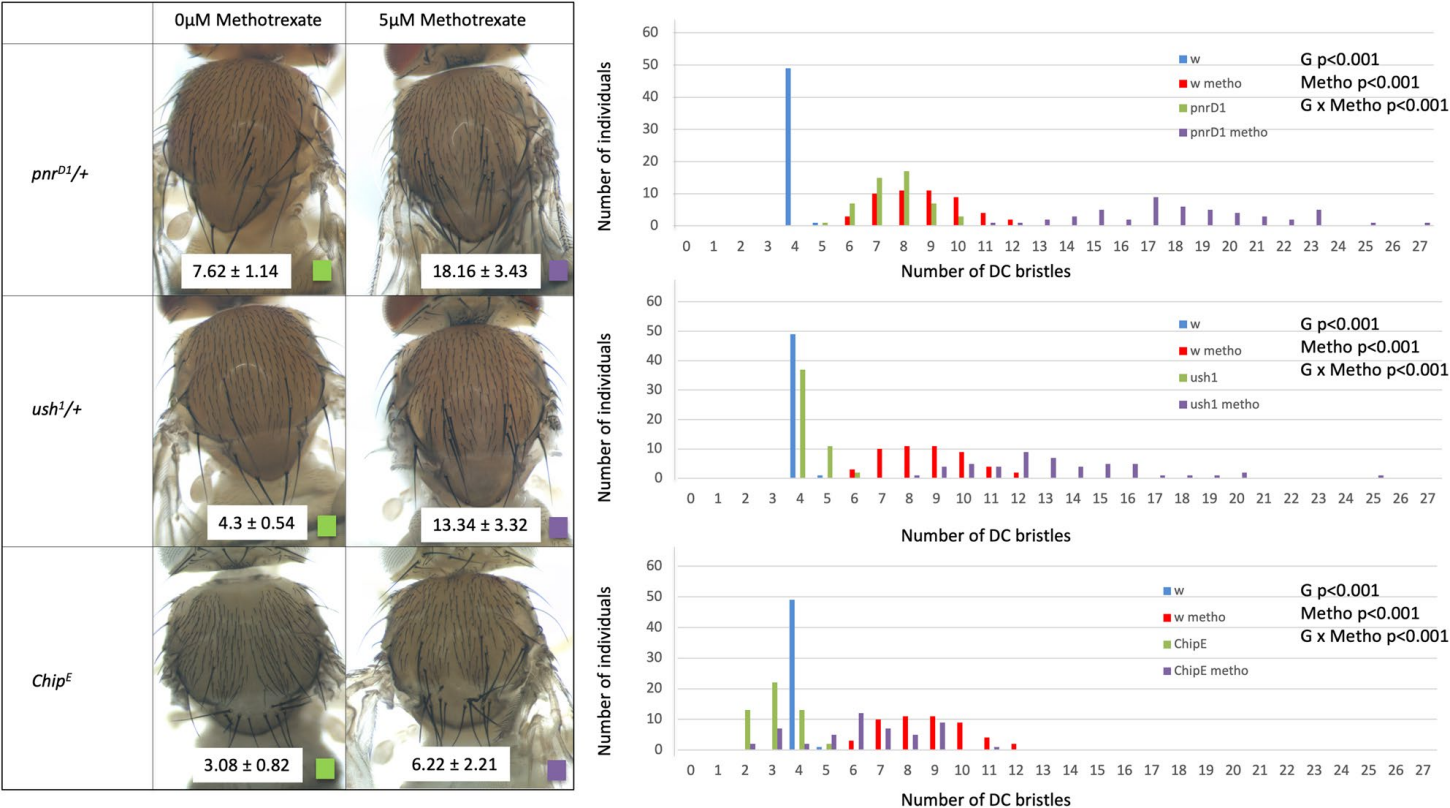
Methotrexate interacts with factors regulating the DC enhancer. Left: DC bristles phenotypes of *pnr*^*D1*^*/* + (top), *ush*^*1*^*/* + (middle) and *Chip*^*E*^ (bottom) mutants grown on standard medium (left) or on 5 µM methotrexate (right). These must be compared to wild-type *w*^*1118*^ grown in the same conditions (Fig. 1). Mean DC bristle number and standard deviations are indicated on a white background on the pictures (N = 50 for each condition). Right: graphical representation of the data (number of individuals per number of DC bristles for each condition).

We tested the effect of a gain of function allele of *pnr*, the allele *pnr*^*D1*^, in heterozygous flies (Fig. 3). This dominant allele carries a missense mutation preventing Pnr interaction with Ush without affecting the DNA binding activity of Pnr, which leads to an over-activation of the DC enhancer^20,27^. *pnr*^*D1*^*/* + flies carry 7.62 ± 1.14 DC bristles (N = 50) when grown on standard medium and 18.16 ± 3.44 DC bristles (N = 50) when grown on 5 µM methotrexate. When compared with wild-type flies grown on standard medium or on 5 µM methotrexate, these data reveal a significant genotype by environment interaction between *pnr* and methotrexate (ANOVA of Aligned Rank Transformed Data, Supplementary file 1, *p* < 0.001). Similar genotype by environment interaction was observed between *u-shaped* and methotrexate. We used *ush*^*1*^, an amorphic allele of *ush*^41^*,* in the heterozygous condition. Reducing *ush* dose by half, using the amorphic allele *ush*^*1*^, had little effect on DC bristle number, 4.3 ± 0.54 DC bristles (N = 50). This number was dramatically increased when *ush*^*1*^*/* + flies was grown on 5 µM methotrexate as flies carry 13.34 ± 3.32 DC bristles (N = 50). When compared with wild-type flies grown on standard medium or on 5 µM methotrexate, this reveals a significant genotype by environment interaction (ANOVA of Aligned Rank Transformed Data, Supplementary file 1, *p* < 0.001). These results shows that the allele *pnr*^*D1*^ and the reduction in *ush* dose by half strongly increase the effect of methotrexate on DC bristle development.

To test for a potential genotype by environment interaction between Chip and methotrexate, we used the *Chip*^*E*^ allele. This homozygous viable allele carries a missense mutation that prevents Chip from interacting with Ac, Sc and Da but not with Pnr^28^. *Chip*^*E*^ reduces the number of DC bristles in flies grown on standard medium (3.08 ± 0.83 DC bristles, N = 50) as previously reported^28^. Accordingly, the effect of methotrexate (5 µM) on bristle number was reduced in *Chip*^*E*^ flies from 8.66 ± 1.53 to 6.22 ± 2.20 DC bristles (N = 50). When compared with wild-type flies grown on standard medium or on 5 µM methotrexate, this reveals a significant genotype by environment interaction (ANOVA of Aligned Rank Transformed Data, Supplementary file 1, *p* < 0.001). Thus, *Chip*^*E*^ strongly reduces the effect of methotrexate on DC bristle number.

In conclusion, the mutants *pnr*^*D1*^ and *ush*^*1*^ that increase the activity of the DC enhancer synergistically interact with methotrexate, whereas the *Chip*^*E*^ mutant, decreasing its activity, strongly reduce the effect of methotrexate on DC bristle development. This suggest that methotrexate might affect the patterning of DC bristles via the activity of the DC enhancer.

### Methotrexate extends the zone of activity of the DC enhancer

**Fig. 4:**
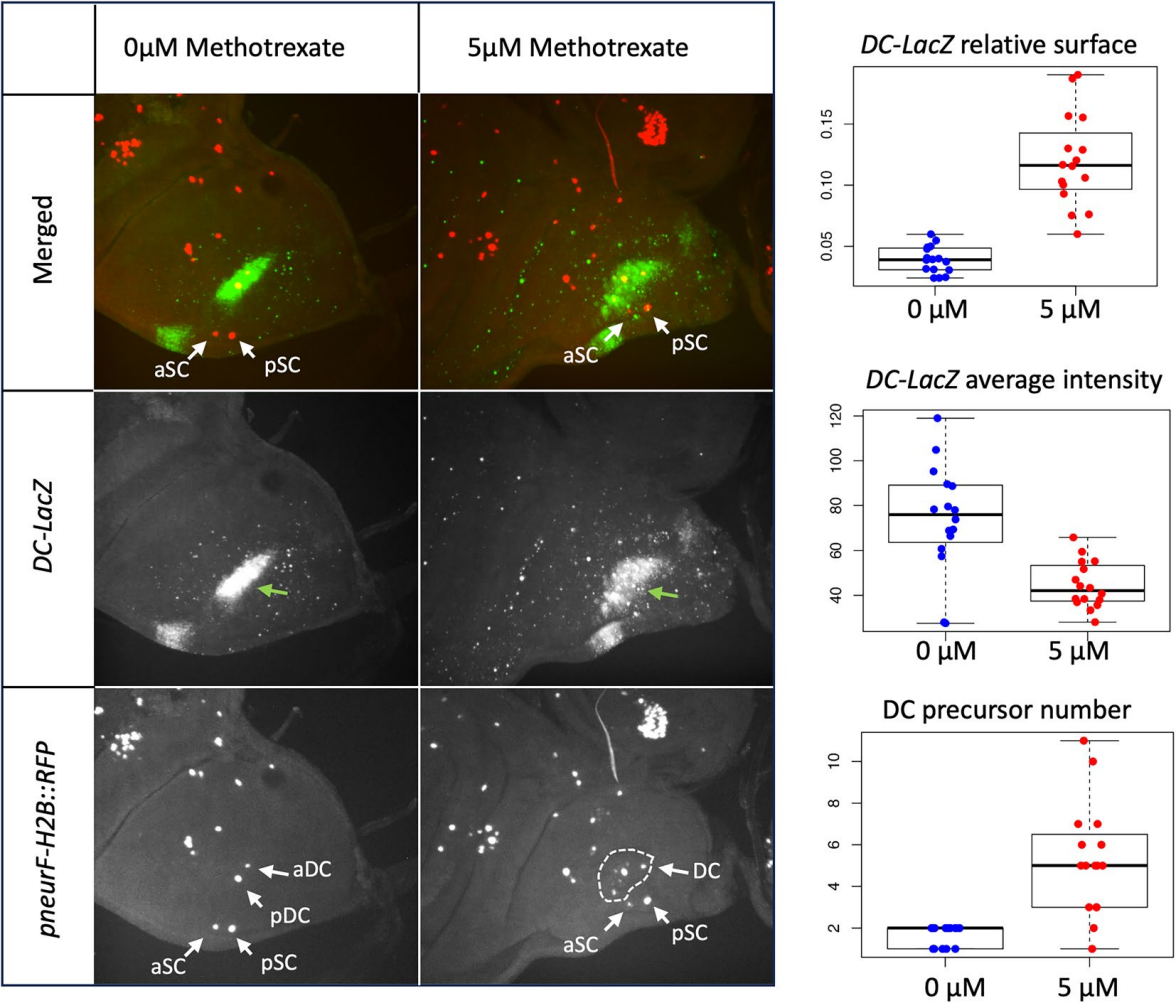
Methotrexate extends the zone of activity of the DC enhancer. *Left*: Visualization of bristle precursors (*pneur-H2B::RFP*, red in merge) and DC enhancer activity (*DC-LacZ*, green in merge) in the presumptive notum of third instar larvae grown on standard medium or 5 µM methotrexate. DC and scutellar bristle precursors are indicated by white arrows (aSC: anterior scutellar, pDC: posterior scutellar, aDC: anterior dorsocentral, pDC: posterior dorsocentral). The multiple precursors formed in the DC region on 5 µM methotrexate are surrounded by a white dashed line. The zone of activity of the DC enhancer is indicated by a green arrow. Right: quantification of the zone of DC activity relative surface (surface stained divided by the square of the presumptive notum width, to take into account size variation of individual wing imaginal discs), the average intensity of the LacZ immunostaining in the DC region and the number of DC bristle precursors, on 0 or 5 µM methotrexate (N = 16 for each condition, *p* < 0.001 in all cases).

In order to visualize the activity of the DC enhancer and test whether it was affected by methotrexate, we used a *lacZ* reporter transgene containing a minimal *hsp70* promoter fused to a 1.4Kb fragment of the *ac-sc* complex containing the DC enhancer^26^. In this line, *lacZ* expression in the DC proneural cluster was previously shown to precisely colocalize with that of *sc*^26^. To visualize bristle precursors we used a *pneurD::H2B-RFP* reporter transgene in which *Histone 2B* gene fused to *RFP* is expressed under the control of *neuralized* regulatory sequences (Fig. 4). The immunostainings and their quantifications revealed that in the wing imaginal disc of L3 wandering larvae grown on 5 µM methotrexate, the zone of activity of the DC enhancer (normalized to the size of the presumptive notum) is larger than in controls (0.12 ± 0.04 versus 0.04 ± 0.01 *p* < 0.001, Fig. 4). Notably, the zone of activity of the DC enhancer extends in the direction of the anterior scutellar bristles (Fig. 4, aSC, arrow). However, in larvae grown on methotrexate containing medium, the average activity of the DC enhancer, as evaluated by the intensity of the staining, appears weaker than on standard medium (44.42 ± 10.43 versus 74.08 ± 24.21, *p* < 0.001, Fig. 4). It is more diffuse, but also more heterogeneous. Indeed, some cells reach a high level of DC enhancer activity, whereas in others the intensity of the staining is weaker than on standard medium. The enlarged zone of activity of the DC enhancer in larvae grown on 5 µM methotrexate includes many ectopic bristle precursor cells (*p* < 0.001, Fig. 4). Indeed, there are in average 5.3 ± 2.7 bristle precursors in presumptive heminotum on 5 µM methotrexate versus 1.6 ± 0.5 on standard medium (*p* < 0.001). The precursor of the anterior DC bristle is known to form later than that of the posterior DC, sometimes after puparium formation in standard conditions^42^. This is why only one DC bristle precursor is observed is some cases on standard medium.

Thus, methotrexate appears to have an effect on DC bristle development via the enlargement of the zone of activity of the DC enhancer.

### Inhibition of Notch signalling by methotrexate is unlikely to contribute to induction of ectopic DC bristle by methotrexate

Because methotrexate induces also the formation of ectopic bristle outside the DC area (Fig. 1, asterisk), although to a lower extent, we wondered whether it could also affect another process. It was shown recently that, in vertebrates, methotrexate reduces Notch (N) signalling^43^. The same effect in flies could reduce lateral inhibition and lead to the formation of ectopic bristles. This led us to test whether manipulation of Notch signalling could modify the impact of methotrexate on DC bristle patterning. To do so we used both a loss of function allele of *Notch* at the heterozygous state, *N*^*55e11*^*,* and a homozygous viable gain of function allele of *Notch*, *N*^*Ax16*^*,*^44,45^.

**Fig. 5:**
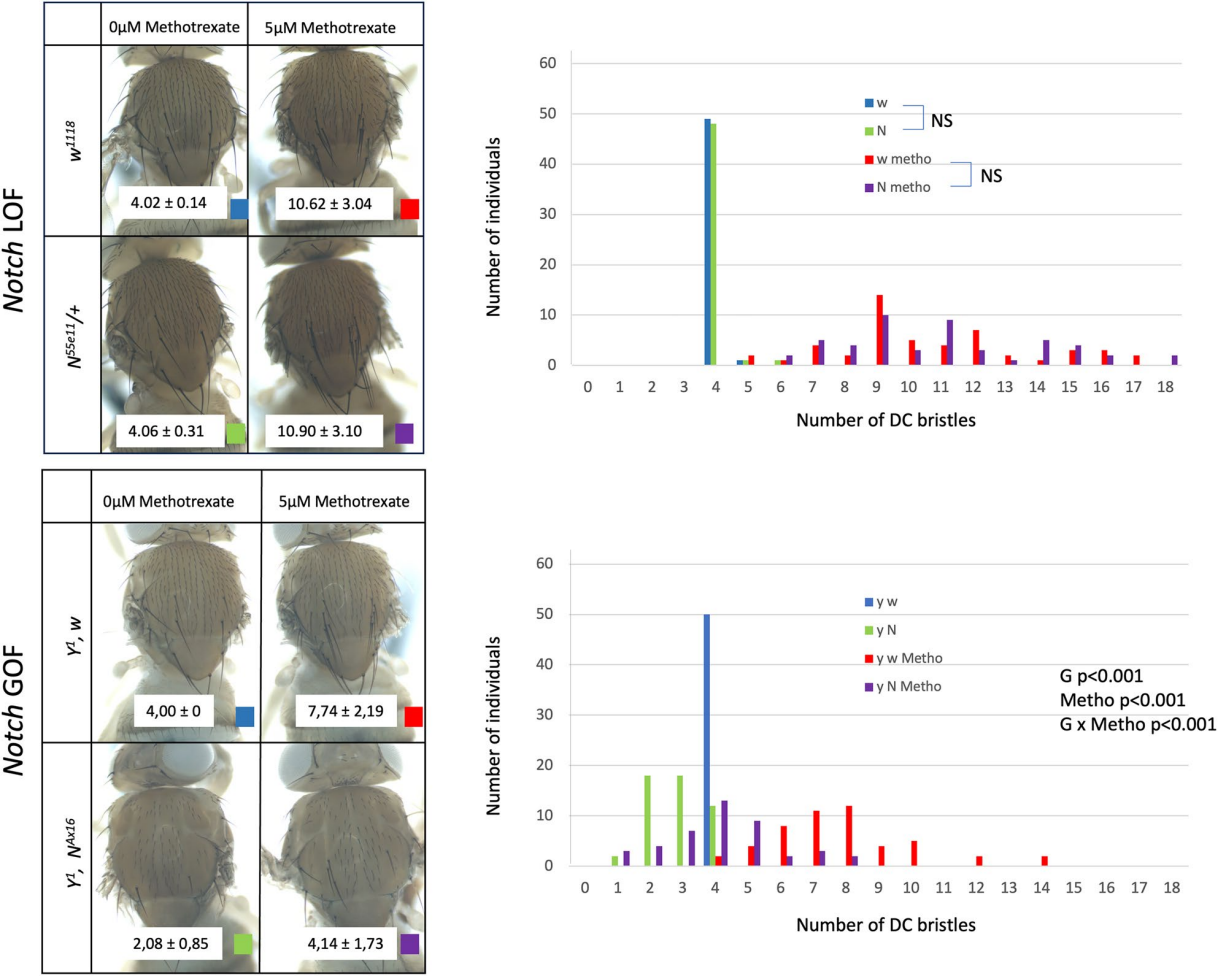
Methotrexate is unlikely to affect DC bristle patterning via Notch signalling A loss of function allele of Notch, *N*^*55e11*^*,* (upper part) and a gain of function allele of Notch, *N*^*Ax16*^, (lower part) were used to test for interaction between Notch signalling and methotrexate for DC bristle patterning. Individuals of the different genotypes were grown on standard medium or on 5 µM methotrexate. Left part: observed phenotypes. Mean DC bristle number and standard deviations are indicated on a white background on the pictures (N = 50 for all conditions except for *N*^*Ax16*^ grown on 5 µM methotrexate for which we obtained only 43 individuals). Right: graphical representation of the data (number of individuals per number of DC bristles for each condition). NS, not significant.

However, for the loss of function allele *N*^*55e11*^, the effect is very weak (Fig. 5) and when *N*^*55e11*^*/* + and *w*^*1118*^ are compared for 0 or 5 µM methotrexate, the differences between genotypes are not significant: 4.06 ± 0.31 versus 4.02 ± 0.14 DC bristles for 0 µM methotrexate (Wilcoxon rank sum test *p* = 0.56) and 10.90 ± 3.10 versus 10.62 ± 3.04 DC bristles for 5 µM methotrexate (Wilcoxon rank sum test *p* = 0.786). This is very different from the strong synergy described previously between methotrexate and *pnr*^*D1*^ or *ush*^*1*^. Thus, we concluded that no synergy is observed between methotrexate and *N* loss of function for DC bristle patterning. In contrast, the gain of function allele *N*^*Ax16*^ significantly interacts with methotrexate (ANOVA of Aligned Rank Transformed Data, Supplementary file 1, *p* < 0.001). It reduces the number of DC bristles on standard medium as previously reported^45^ (2.08 ± 0.85 versus 4.00 ± 0 DC bristles) and the effect of methotrexate on DC bristle number (4.14 ± 1.73 versus 7.74 ± 2.19 DC bristles). Nevertheless, since lateral inhibition takes place after the induction of *ac-sc* expression in proneural cluster by pattering factors, it is expected that increasing lateral inhibition with a gain of function allele of Notch reduces the effect of methotrexate which acts on the DC enhancer. Thus, regarding induction of ectopic DC bristle, these experiments do not suggest a major role of methotrexate via Notch signalling.

### Genes encoding repressors of *ac-sc* basal expression and temperature show genotype by environment interactions

**Fig. 6:**
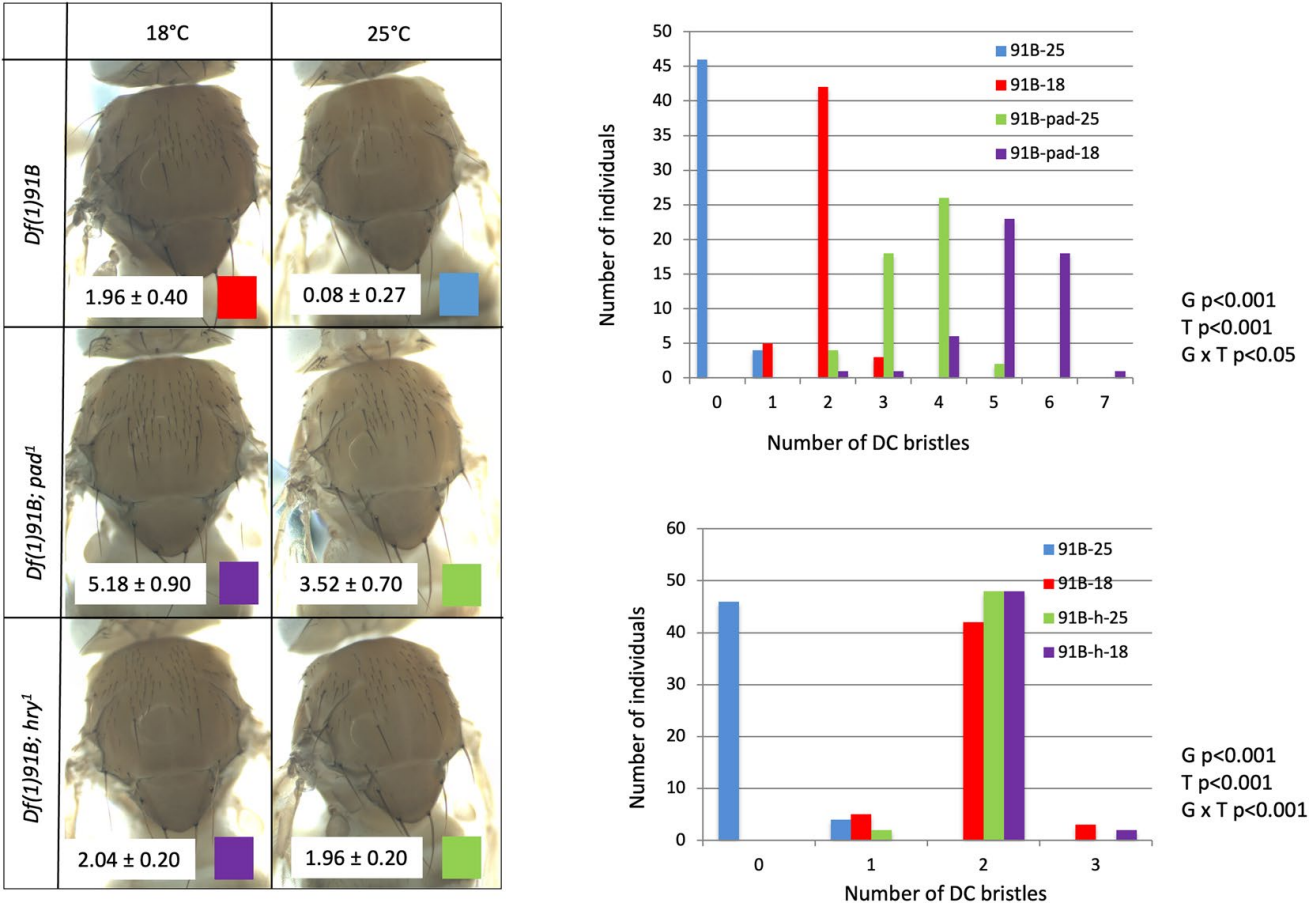
Temperature interacts with repressors of *ac-sc* basal expression. Left: DC bristle phenotypes of *Df(1)91B*, *Df(1)91B; pad*^*1*^ and *Df(1)91B; h*^*1*^ grown at 18 °C (left) or 25 °C (right). Mean DC bristle number and standard deviations are indicated on a white background on the pictures (N = 50 for each condition). Right: graphical representation of the data (number of individuals per number of DC bristles for each condition).

The ectopic bristles observed at low temperature anteriorly to the DC bristles are reminiscent of some of the ectopic bristles induced by mutations in repressors of *ac-sc* basal level^30,31^*.* An impact of low temperature on this repressive system would explain why low temperature is able to induce the development of DC bristles in the absence of the DC enhancer. Thus, we tested for interactions between temperature and alleles of two of these repressors, *hairy*, (*hry)* and *poils au dos,* (*pad)*. We chose *hry*^*1*^*,* a hypomorphic homozygous viable allele caused by a *gypsy* insertion 5kb upstream of the promoter^46^, and *pad*^*1*^*,* encoding a truncated protein and also homozyogous viable^30^. We performed these tests in the *Df(1)91B* background (Fig. 6). This allowed to analyse potential genotype by environment interactions between temperature and the repressive system without the confounding effect of the DC enhancer.

The number of DC bristles in *Df(1)91B; pad*^*1*^ flies is strongly increased compared to *Df(1)91B* flies at both 18 °C and 25 °C: 5.18 ± 0.90 DC bristles at 18 °C (purple, N = 50) and 3.52 ± 0.70 DC bristles at 25 °C for *Df(1)91B; pad*^*1*^ flies (green, N = 50; Fig. 6), but the difference with *Df(1)91B* flies is higher at 25 °C. This reveals a significant genotype by interaction between temperature and *pad* in the *Df(1)91B* background (ANOVA of Aligned Rank Transformed Data, Supplementary file 1, *p* < 0.05). This suggests that *pad* is involved in the effect of temperature on DC bristle patterning.

At 25 °C, *Df(1)91B; hry*^*1*^ flies carry 1.96 ± 0.20 DC bristles (green, N = 50), whereas most *Df(1)91B* flies have no DC bristles (0.08 ± 0.27 DC bristles, N = 50, blue, Fig. 6). At 18 °C no significant difference is observed between the two genotypes: 1.96 ± 0.40 DC bristles for *Df(1)91B* flies (red, N = 50) and 2.04 ± 0.20 DC bristles for *Df(1)91B; hry*^*1*^ flies (purple, N = 50). This reveals a strong genotype by environment interaction between *hry* and temperature for DC bristle number in the *Df(1)91B* background (ANOVA of Aligned Rank Transformed Data, Supplementary file 1, *p* < 0.001). This suggests that the *hry*^*1*^ mutation strongly affects the mechanisms by which temperature modulates the patterning of DC bristle. In particular, our experiments show that *hry* is required for the repression of DC bristle development at 25 °C in the *Df(1)91B* background.

Thus, the experiments with *hry* and *pad* mutants show that temperature interact with repressors of *ac-sc* basal level.

## Discussion

Our experiments show that methotrexate increases DC bristle number through the DC enhancer. Indeed, in flies deleted for this enhancer, methotrexate does not induce ectopic bristles. Furthermore, methotrexate shows strong gene by environment interactions with factors regulating the DC enhancer and, moreover, it extends the zone of activity of this enhancer. The precursors of ectopic bristles form within this enlarged zone of activity. In the zone of activity of the DC enhancer, the selection of bristle precursors is mediated by lateral inhibition through Notch signalling, which is known to be modulated by methotrexate in vertebrates^43^. Nevertheless, our results do not support an impact of methotrexate on Notch signalling for DC bristle patterning, although we cannot exclude that the genetic condition we used for Notch loss of function (heterozygosity for the allele *N*^*55e11*^) is not strong enough. In any case, our data suggest that the enlarged zone of activity of the DC enhancer induced by methotrexate likely gives enough space for lateral inhibition to restrict *ac-sc* expression to a higher number of bristle precursors, whereas on standard medium, its smaller size is compatible with the emergence of only two bristle precursors even if the average activity of the DC enhancer is higher.

Few methotrexate direct targets have been identified. This drug is a folate analogue that targets the dihydrofolate reductase, an enzyme involved in purine metabolism^35^. Thus, methotrexate affects DNA replication, hence its use in cancer treatment. However, it also affects particular developmental pathways such as JAK-STAT signalling^43,47^. In particular, the impact of methotrexate on JAK-STAT signalling is thought to explain its effectiveness in treating rheumatoid arthritis^48^. Transcriptomes of adult *Drosophila* females fed with methotrexate, ovaries from such females and *Drosophila* cell lines treated with methotrexate have been analysed^49^. However, among the genes whose expression was modulated by methotrexate we identified no obvious candidate that could participate to the DC bristle phenotype, probably because the transcriptomes were not analysed in the presumptive mesonotum. In addition, other genes involved in DC development could be analysed to test their interaction with methotrexate. For example, *wingless* (*wg*), which is expressed in an antero-posterior stripe in the presumptive notum in the wing imaginal disc, plays a permissive role in DC enhancer activity^26^. This is mediated by the inhibition of the kinase Shaggy (Sgg) by Wg signalling^50^. Indeed, Sgg phosphorylates Pnr and Sc, reducing their proneural potential. Thus, the inhibition of Sgg by Wg signalling promotes DC bristle formation. Given the strong interaction between *pnr* and methotrexate that we observed, it would be interesting to investigate a potential interaction between *Sgg* and this drug. Further work is needed to understand the strong impact of methotrexate on DC enhancer activity, but this strong and specific effect suggests that this drug found as pollutant in waste waters could affect particular developmental processes in wild life.

In contrast, to methotrexate, low temperature is able to induce DC bristles in the region of the anterior DC bristles in absence of the DC enhancer. Our results suggest that this is because temperature affects *ac-sc* regulation through another mechanism. Indeed, temperature interacts with repressors of *ac-sc* basal expression, in particular with *hry* and to a lesser extent *pad*. Hry was shown to be a direct transcriptional repressor of *ac-sc*^32^. Moreover, *pad* loss of function increases *ac* and *sc* expression in a region anterior to the DC domain^30^ where extra bristles are induced by cold. Thus, we favour the hypothesis that cold induces ectopic DC bristles by up-regulating the basal expression of *ac-sc*.

QTL studies have shown that there is genetic variation at *hry* in natural populations of *Drosophila melanogaster* that affects the number of particular bristles^51^ although this was not replicated in a second study using wild-caught flies, perhaps because of epistasis or environmental effects^52^. This suggests that variation in environmental temperature could facilitate the evolution of DC bristle pattern by revealing cryptic genetic variation in loci such as *hry*. We do not know how temperature could affect the regulation of *ac-sc* basal expression by the cocktail of repressor. However, some experiments on redundant enhancers (shadow enhancers) have shown that high temperature weakens the activity of certain enhancers^53,54^, which makes conceivable that low temperature reinforces the activity of particular regulatory sequences. For instance, *ac-sc* repression by Hry might be weaker at low temperature.

In this study, we extensively employed the strategy of statistically supported genotype-by-environment interactions. When it is statistically significant, it shows that the phenotypic differences between mutants and controls are affected by the environment. Conversely, it reveals that the phenotypic impact of the environment depends on allelic variation of the tested gene. This approach shows that the tested gene is directly or indirectly involved in the effect of the environment without providing a molecular mechanism of action. Knowledge of the mode of action of the tested gene, as well as complementary functional data, are required to gain mechanistic insights. Thus, our data shows that the action of methotrexate on DC bristle patterning takes place via the DC enhancer. We demonstrate interactions between this drug and the DC enhancer regulators *pnr*, *ush* and *Chip*, but we also show the requirement of the DC enhancer for the effect of this drug (using the *Df(1)91B* background), as well as the modulation of the DC enhancer reporter transgene by this drug. We also show that cold-induced ectopic bristles were observed in the absence of the DC enhancer, and genotype-by-environment interactions were demonstrated between temperature and genes *hry* and *pad*, which encode two known repressors of *ac-sc* basal expression. Although this shows that cold exerts its effect on DC bristle patterning independently of the DC enhancer, additional functional experiments are required to validate the hypothesis that cold increases *ac-sc* basal expression.

Taken together, our results show that methotrexate and low temperature both induce ectopic DC bristles through the regulation of *ac-sc* proneural genes but by distinct mechanisms: methotrexate through the DC enhancer and cold independently of this enhancer, by interacting with repressors of *ac-sc* basal expression. This shows the complexity of the interaction between a developing organism and its environment, with different factors of the exposome targeting the same master genes via distinct regulatory mechanisms.

## Methods

### Medium preparation

Standard medium was composed of: 7.5% yeast, 9% cornmeal, 1% agar and 0.27% Moldex. 5 µM methotrexate was added after cooking before distributing the medium into vials. Experiments with methotrexate were performed on several batches of medium and mutants were always compared to controls developed in tubes from the same batch.

### Fly stocks

*W*^*1118*^ or *y*^*1*^*w*^*1118*^ were used as wild-type. The stocks *Df(1)91B* and *pad*^*1*^ were previously described^30^. The following stocks were ordered to Bloomington Drosophila Stock Centre:

- *Kr*^*If-1*^*/In(2LR)Gla, wg*^*Gla-1*^* PPO1*^*Bc*^*; P{ry[*+ *t7.2]* = *ac-lacZ.AS1.4DC}3, ry*^*506*^ (aka *DC-LacZ*, BL-36557)
- *mwh*^*1*^* jv*^*1*^* pnr*^*D1*^*/TM2* (BL-36551)
- *ush*^*1*^* cn*^*1*^* bw*^*1*^* speck*^*1*^*/CyO* (BL-35503)
- *w*^*1118*^*; Chip*^*E*^ (BL-36527)
- *hry*^*1*^ (BL-513)
- *N*^*55e11*^*/FM7c* (BL-28813)
- *y*^*1*^,w*, N^*Ax16*^ (BL-52813)
- *pneurD::H2B-RFP* is a gift from François Schweisguth.

Adult flies were allowed to lay eggs for 3–5 days on the different media, and development from embryo to adult took place on these media. Thus, the different media (standard or containing methotrexate) were consumed by the larvae during their whole development.

### Immunostainings

Larvae were dissected inside-out in PBS. They were fixed 10 min in 4% paraformaldehyde in PBS, washed thrice 5 min in PBT (PBS, 0.1% Triton). They were then incubated in blocking solution (1% BSA in PBT) for 1 h, incubated 1 h with Rabbit anti-Beta-Galactosidase (Cappel Lab, 1/1000 dilution) and washed thrice in PBT 1% BSA. After this, they were incubated with Rat anti-RFP (5F8, Chromoteck, 1/500 dilution) for 1 h, washed thrice in PBT 1% BSA. They were then incubated for 1 h with fluorochrome conjugated antibodies (Molecular probes, 1/1000 dilution) and then washed thrice 5 min in PBT. Wing imaginal discs were then dissected and mounted.

### Thorax photographs

Adult thoraces were imaged with a dissecting microscope equipped with a Leica DC480 digital camera using the Leica IM50 Image Manager software.

### Microscopy

Images were acquired with an Olympus BX-41 microscope using the metaview software. Images were treated with ImageJ and Photoshop.

### Statistics

DC bristles were counted in 50 females for each condition but *N*^*Ax16*^ flies grown on 5 µM methotrexate for which we obtained only 43 females. Files with row data for the different figures are provided as supplementary material (Row data for Figs. 1, 2, 3, 4, 5 and 6). Statistical analyses were performed in Excel or R Studio. We used Wilcoxon Ranked Sum test to compare two conditions and ANOVA on Aligned Rank Transformed Data (ARTOOL package) to test for interactions between genotypes and either methotrexate or temperature (Supplementary file 1). When experiments with different alleles were performed simultaneously, the same genetic controls (either *w*^*1118*^ or *Df(1)91B*) were used for different tests. Student’s t-test were performed to compare the relative surfaces of the zone of activity of the DC enhancer, the average intensity of the DC-LacZ immunostaining and bristle precursor number (Fig. 4, Raw data for Fig. 4).

## Supplementary Information

Supplementary Meterial 1.

Supplementary Information 2.

Supplementary Information 3.

Supplementary Information 4.

Supplementary Information 5.

Supplementary Information 6.

Supplementary Information 7.

Supplementary Information 8.

## Data Availability

Raw data are available as supplementary material (Raw data for Fig. 1–6).
