# Supplementary material for: Exposome variations affect Drosophila bristle patterning via the regulation of proneural genes through distinct mechanisms
Source: Sci Rep. 2025 Oct 16;15:36234. doi: 10.1038/s41598-025-20122-6 (PMC12533043; doi:10.1038/s41598-025-20122-6)
Supplement: Supplementary file 1 — Supplementary Meterial 1. [file 41598_2025_20122_MOESM1_ESM.docx]

**Supplementary file 1:** Tables of the Anova on aligned rank transformed data for figure 3, 5, 6.

**Row data for Figure 1-6:** Files with row data corresponding to Figure 1-6
