# Supplementary material for: Exposome variations affect Drosophila bristle patterning via the regulation of proneural genes through distinct mechanisms
Source: Sci Rep. 2025 Oct 16;15:36234. doi: 10.1038/s41598-025-20122-6 (PMC12533043; doi:10.1038/s41598-025-20122-6)
Supplement: Supplementary file 2 — Supplementary Information 2. [file 41598_2025_20122_MOESM2_ESM.docx]

Anova on aligned rank transformed data for figure 3, 5, 6

Anova on aligned rank transformed data:

Variable: DC bristle number.

Factors: Genotype (*WT* or *pnr^D1^)* and methotrexate (0µM or 5µM)

|  | Df | Df.res | F value | Pr (>F) |
| --- | --- | --- | --- | --- |
| Genotype | 1 | 196 | 530.00 | <2.22E-16 |
| Metho | 1 | 196 | 616.73 | <2.22E-16 |
| Genotype x Metho | 1 | 196 | 173.65 | <2.22E-16 |

Anova on aligned rank transformed data:

Variable: DC bristle number.

Factors: Genotype (*WT* or *ush^1^)* and methotrexate (0µM or 5µM)

|  | Df | Df.res | F value | Pr (>F) |
| --- | --- | --- | --- | --- |
| Genotype | 1 | 196 | 131.66 | <2.22E-16 |
| Metho | 1 | 196 | 712.59 | <2.22E-16 |
| Genotype x Metho | 1 | 196 | 116.20 | <2.22E-16 |

Anova on aligned rank transformed data:

Variable: DC bristle number.

Factors: Genotype (*WT* or *chip^E^)* and methotrexate (0µM or 5µM)

|  | Df | Df.res | F value | Pr (>F) |
| --- | --- | --- | --- | --- |
| Genotype | 1 | 196 | 95.752 | <2.22E-16 |
| Metho | 1 | 196 | 426.891 | <2.22E-16 |
| Genotype x Metho | 1 | 196 | 15.932 | 9.2753E-05 |

Anova on aligned rank transformed data:

Variable: DC bristle number.

Factors: Genotype (*WT* or *N^Ax16^)* and methotrexate (0µM or 5µM)

|  | Df | Df.res | F value | Pr (>F) |
| --- | --- | --- | --- | --- |
| Genotype | 1 | 189 | 164.769 | <2.22E-16 |
| Metho | 1 | 189 | 189.772 | <2.22E-16 |
| Genotype x Metho | 1 | 189 | 60.795 | 4.1419E-13 |

Anova on aligned rank transformed data:

Variable: DC bristle number.

Factors: Genotype (*Df(1)91B* or *Df(1)91B; pad^1^)* and Temperature (18°C or 25°C)

|  | Df | Df.res | F value | Pr (>F) |
| --- | --- | --- | --- | --- |
| Genotype | 1 | 196 | 806.45 | <2E-16 |
| Temperature | 1 | 196 | 464.0731 | <2E-16 |
| Genotype x Temperature | 1 | 196 | 5.3539 | 0.021713 |

Anova on aligned rank transformed data:

Variable: DC bristle number.

Factors: Genotype (*Df(1)91B* or *Df(1)91B; hry^1^)* and Temperature (18°C or 25°C)

|  | Df | Df.res | F value | Pr (>F) |
| --- | --- | --- | --- | --- |
| Genotype | 1 | 196 | 660.35 | <2.22E-16 |
| Temperature | 1 | 196 | 770.94 | <2.22E-16 |
| Genotype x Temperature | 1 | 196 | 618.07 | <2.22E-16 |
